# A Pathway for Improving Performance and Interpretation of Strain in a Pediatric Echocardiography Laboratory

**DOI:** 10.1111/echo.70369

**Published:** 2025-12-11

**Authors:** Shivani Patel, Nazia Husain, Jennifer Acevedo, Stefani Samples, Amanda Hauck

**Affiliations:** ^1^ Ann and Robert H. Lurie Children's Hospital of Chicago Chicago Illinois USA; ^2^ Northwestern University Feinberg School of Medicine Chicago Illinois USA

**Keywords:** echo education, pediatric strain, quality improvement

## Abstract

**Background:**

Echocardiographic quantification of myocardial deformation is a valuable tool to assess left ventricular systolic function in children at risk for systolic dysfunction. Since clinical utility is dependent on accurate and reproducible data, pediatric echo labs should establish a process to ensure high quality strain performance and interpretation. We describe our institutional experience of a successful strain implementation pathway.

**Methods:**

Starting in 2016, various plan‐do‐study‐act cycles were implemented, including a comprehensive echocardiography function protocol, knowledge and skill‐based education, updated equipment and software to optimize acquisition, reporting, and billing for global longitudinal strain (GLS). We reviewed a sample of echocardiograms annually from 2019 to 2023 on children at risk for chemotherapy related cardiotoxicity (CTRCD). We calculated the percentage of echocardiograms that performed and reported GLS, reviewing for accuracy.

**Results:**

From 2019 to 2023, 685 echocardiograms were reviewed. GLS reporting increased from 39% in 2019 to 76% in 2023 and accuracy improved from 57% to 72% (*p* < 0.001). Reasons for inaccurate GLS included poor 2D image quality (24%), endocardial border tracing errors despite good image quality (45%) or both (21%). In almost half the studies where GLS was measurable but not reported [99/214; 46%], GLS had not been performed on the echo cart. Reporting of GLS improved from 27% in 2019 to 66% in 2023 with on‐cart automation and easier post‐processing; and other interventions, including iterative education.

**Conclusions:**

Implementation of administrative, technological, and educational interventions helps to establish a pathway of consistent, high‐quality performance and accurate reporting of GLS in pediatric echocardiography labs.

## Graphical abstract

The process map shows how implementation of administrative, technological, and educational interventions helps to establish a pathway for high‐quality performance and accurate reporting of GLS in a pediatric echocardiography lab. Strain automation, integration of vendor‐neutral post‐processing platform into reporting software, and iterative education are key in this process.

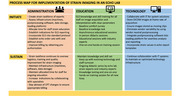

**Abbreviations**
CPT: current procedural terminology
CTRCD: chemotherapy related cardiotoxicity
EF: ejection fraction
GLS: Global Longitudinal Strain
LV: left ventricular
PDSA: plan‐do‐study‐act

## 1 Introduction

Echocardiographic quantification of myocardial deformation has emerged as a valuable tool to assess left ventricular (LV) systolic function in children at risk for systolic dysfunction [1, 2]. Strain‐based imaging techniques, specifically 2‐dimensional speckle tracking strain is an increasingly used echocardiographic technology that provides clinical utility in a variety of conditions and has been validated in healthy pediatric populations [3, 4, 5, 6, 7, 8]. Strain is sensitive to subclinical disease, more reproducible than ejection fraction, is a better measure of myocyte function, and can assess regional function [9]. Children at risk for cardiac dysfunction secondary to chemotherapy related cardiotoxicity (CTRCD) are a representative group in whom myocardial strain imaging detects subclinical cardiac dysfunction and is being widely applied in clinical practice [10, 11, 12, 13]. The American Society of Echocardiography guideline paper on performing a comprehensive pediatric transthoracic echocardiogram recommends the incorporation of LV GLS into LV functional protocols [14].

**FIGURE 1:**
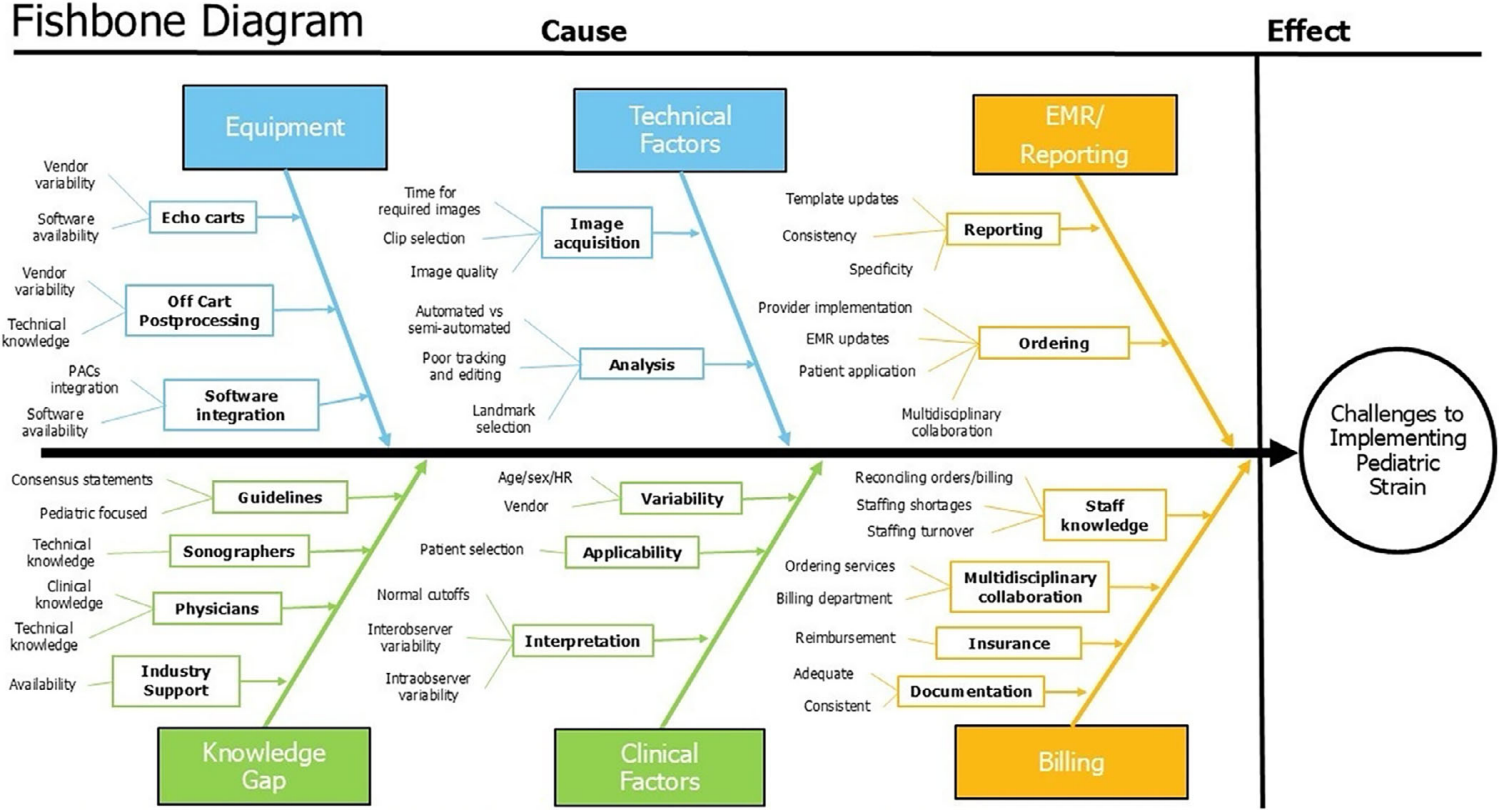
Fishbone diagram depicting causes of challenges for implementing GLS in a pediatric echocardiography laboratory. Blue boxes represent technical factors, orange boxes represent administrative factors, and green boxes represent education/clinical factors.

The clinical utility of this modality is dependent on accurate and reproducible data; therefore, pediatric echo labs must establish a pathway to ensure high quality strain imaging and interpretation. There are several technical and clinical factors that affect the incorporation of accurate strain into a pediatric echocardiography lab, ranging from personnel buy‐in for newer technologies, variability in the acquisition and interpretation of strain, and barriers to reporting [15]. Furthermore, in pediatric echocardiography, apical images differ from adult protocols in their “upside‐down” orientation, limiting the use of echo machines’ standard inbuilt algorithms. Standard algorithms must be adjusted to align images appropriately for strain software to be used effectively. Additionally, high heart rates in pediatric patients result in relatively low frame rates which may preclude effective acquisition and storage of images for strain analysis if not addressed. In our barriers assessment, we highlight these potential factors as reported in the literature as well as from our own experiences implementing global longitudinal strain (GLS) using 2‐dimensional echo speckle tracking in the lab (Figure 1).

Given these challenges in implementing strain, we describe our institutional experience utilizing data from children undergoing pediatric echocardiograms for CTRCD. Our work aims to provide a framework for other pediatric echocardiography labs to overcome these barriers in instituting and maintaining high quality strain imaging and interpretation. We conducted cyclical interventions to improve the reporting and accuracy of strain, with the aim to increase reporting of GLS to at least 80% of the scans and to improve GLS accuracy by 50% over the study period.

## 2 Methods

### 2.1 Context

**FIGURE 2:**
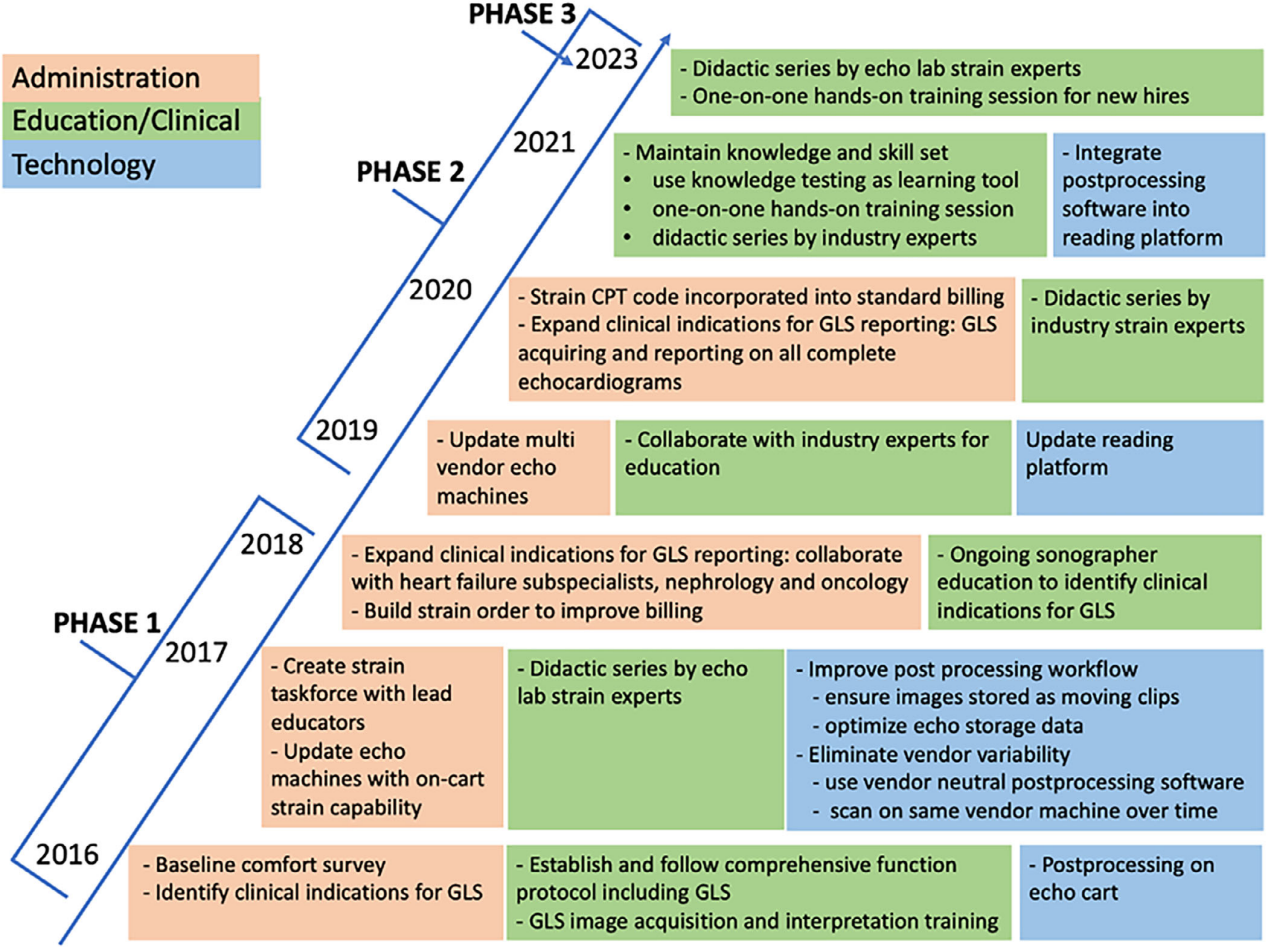
Administrative, educational and technological interventions performed to improve accurate reporting of GLS.

This study reports our experience of implementing strain in our echo lab over a seven‐year period from 2016 to 2023. Phase 1 describes our early implementation of GLS imaging, Phase 2 reviews our effort in improving of GLS imaging using plan‐do‐study‐act (PDSA) cycles and finally Phase 3 is comprehensive evaluation of our performance in acquisition, interpretation, and reporting of GLS. The timelines of our experience with regards to the various administrative, technical, and educational endeavors are depicted in Figure 2.

### 2.2 Phase 1: 2016 to 2019–Implementation of GLS Imaging

In 2016, at a time when the validity and clinical application of GLS in the pediatric population were not well established, we implemented GLS in our lab.

**Administrative planning**: Dedicated education time was built in the echo lab's clinical schedule for the sonographers.

**Technology planning**: GLS was performed using a single vendor echo machine with post‐processing on the echo cart itself.

**Education planning**: Education on GLS image acquisition, interpretation, and reporting was done using in‐person and video recorded training sessions that demonstrated step‐by‐step instructions on how to acquire images, use the semi‐automated strain imaging tool and correct tracings. A comprehensive cardiac function assessment protocol (see Supplement 1) was created that detailed necessary imaging. This included setting baseline requirements for image quality including the presence of a clear ECG tracing with visible P and QRS waves, frame rates of 60–90 fps (at least 2/3rd of patient's heart rate), depth optimized to fill the sector with the ventricle and sector width optimized to include the entire myocardium throughout the cardiac cycle. Automated software defined end diastole and end systole using automated aortic valve closure detection algorithms or peak R wave detection and end of T wave detection respectively. These timings were manually modified in patients with conduction abnormalities. In those, visualization of mitral valve closure and aortic valve closure were used to determine end‐diastole and end‐systole respectively. Our echo lab strain experts provided literature with guidelines on strain imaging and its clinical utility, establishing lab standards for “normal” and “abnormal” GLS based on available literature at the time [3, 16, 17, 18]. A peak systolic GLS value less negative than −18% was considered abnormal for children > 2 years old and was set as the lab standard as a cut off for reporting GLS as abnormal. Values between –17% and –18% were considered borderline.

For this phase, we collected data on a 2016 baseline survey that assessed sonographers and physicians’ comfort in GLS utilization (Supplement 2, Survey 1; Supplement 3, Survey 2). In 2018, with the creation of a strain order, we collected data on the total number of echoes with strain that were billed.

### 2.3 Phase 2. 2019–2023, Improvement of GLS Imaging

**FIGURE 3:**
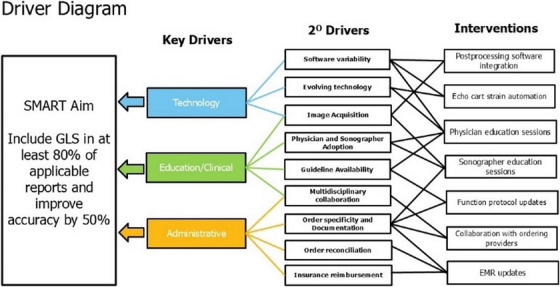
Key driver diagram with SMART aim to implement and improve reporting consistency and accuracy for global longitudinal strain (GLS).

Based on our initial experience and conversations with key stakeholders, including the strain taskforce, we developed a key driver diagram with our SMART aim (Figure 3). This was used to plan and perform various interventions and guided metrics with which to monitor the process.

PDSA cycles were utilized to address various administrative, technical and educational issues; these included streamlining ordering and billing for GLS, technical/equipment updates for acquisition, analysis, interpretation and reporting, and updated comprehensive educational modules for GLS. The educational sessions included one‐on‐one hands‐on skill sessions with a strain educator that included 3 preselected cases of normal and abnormal GLS. These hands‐on sessions supplemented asynchronous and live educational sessions by strain experts. These PDSA cycles and their timelines for all interventions performed are depicted in Figure 2.

During phase 2, data collected included a comfort survey in 2023 that assessed sonographer and physician comfort in GLS utilization. This survey included additional questions to evaluate the unanticipated consequences of implementing strain to utilize as a balancing measure. A knowledge test was administered to each sonographer and physician in the lab that assessed their understanding of strain acquisition and interpretation including the clinical utility of GLS both before (mandatory) and after (optional) the comprehensive educational module.

### 2.4 Phase 3: Comprehensive Evaluation of Performance in Acquisition, Interpretation, and Reporting of GLS

We completed a comprehensive evaluation of our lab's performance in acquisition, interpretation, and reporting of GLS using samples of echocardiograms performed for children with CTRCD in the last quarter of 2019, 2020, 2021, 2022, and 2023. The percentage of CTRCD echocardiograms that included reported GLS was noted. If strain was reported, the echo was reviewed by our strain experts to determine the accuracy of the reported GLS and the reasons for inaccuracies (if any). If strain was not reported, the echo was reviewed to determine whether strain could potentially have been performed accurately. We also reviewed the number of studies that were appropriately reimbursed from 2019 to 2023.

### 2.5 Data Analysis

Comfort surveys completed by sonographers were compared between 2016 and 2023, with no statistical analysis. *t*‐Tests were used to compare the knowledge test scores before and after comprehensive educational modules conducted in 2021. Chi‐square analysis was used to compare the reporting and accuracy of GLS interpretation from 2019 to 2023. Statistical process control charts (*p* chart) were used to visualize changes in reporting and accuracy over the interventions with baseline shifts, and any associated variation. A Pareto chart was used to show the factors that most significantly impacted the accuracy of GLS reporting, with the most significant problems on the left and listed in descending order on the bar graph with the cumulative total on the line graph.

## 3 Results

### 3.1 Phase 1 and 2: Initiation and Improvement of Strain Imaging With PDSA Cycles

The administrative, education, and technology related goals, obstacles, and interventions that were employed in our center are demonstrated in Table 1 below.

**TABLE 1:** Impediments, interventions, and outcomes during strain implementation pathway.

Purpose	Elements	Impediments	Interventions	Outcomes
**Administration** Identify and increase the number of echocardiograms on which GLS is performed, reported, and billed appropriately.	‐Allocate time for education. ‐Assess and maintain comfort. ‐Increase ordering. ‐Set up technical infrastructure. ‐ Set up billing infrastructure.	‐ Staff turnover. ‐ Decreased GLS ordering due to lack of buy‐in and interest from providers. ‐ Cumbersome technology that required repeated manual adjustments, making the process time intensive and less reliable, with significant user variability. ‐ Strain not always included in pre‐authorization process, leading to high rate of insurance denial. ‐ Lack of commensurate reimbursement.	‐ Create strain standardization task force. ‐ Collaborate with ordering providers. ‐ Update on‐cart strain assessment. ‐ Train sonographers to identify GLS indications. ‐ Create a separate strain order in the electronic medical record. ‐ Incorporate GLS imaging into standard echo protocol. ‐Incorporate strain CPT into echo order.	‐ Increased ordering of strain imaging. ‐ Increased identification of patients that needed GLS. ‐ Standard billing practices established
**Technology**: Maintenance of technology upgrades and updates to optimize workflow and efficiency in a multivendor echo lab.	‐ Optimize image acquisition and analysis for GLS. ‐ Optimize accurate GLS interpretation and reporting.	‐ Echo image compression limits post‐processing ‐ Cine images unavailable for physician review ‐ Variation in strain data amongst vendors, limiting trending. ‐ Difficulty scheduling patients on same vendor machine. ‐ Physician review required a separate, remote workstation, interrupting echo lab workflow	‐ Collaborate with IT to optimize data storage. ‐ Attempt to schedule patients on same vendor machine and use of vendor‐neutral post‐processing software for accurate data trending. ‐ Incorporate strain in reporting template, including values and interpretation. ‐ Integrate vendor‐neutral post‐processing software into reading platform.	‐ Elimination of vendor variation in strain reporting ‐ Elimination of interruption in workflow with integration of post‐processing software into reading platform
**Education**: Establish and maintain user knowledge and skill in image acquisition and analysis; interpretation and reporting of GLS.	‐ Establish a comprehensive function protocol. ‐ Education in image acquisition and analysis for multiple vendors. ‐ Education in GLS utility.	‐ Unpredictable staffing turnover. ‐ Newer echo machines from multiple vendors with differences in acquisition. ‐ Rapidly evolving technology for echo machines and vendor‐neutral post‐processing software.	‐ GLS knowledge and skill training for all new staff. ‐ Use of knowledge testing as a learning tool. ‐ Steady didactic series with asynchronous and in‐person sessions by echo lab and industry experts. ‐ One on one hands‐on skills training session with strain expert.	‐ Ongoing educational sessions ‐ Onboarding of new staff includes strain training

Sequential comfort surveys demonstrated that between 2016 and 2023, there was an increase in the sonographers’ perceived ability to recognize the need for strain imaging in every patient (increased from 22% to 48%), feeling very comfortable with image acquisition (increased from 33% to 73%), and recognizing a normal GLS value (increased from 56% to 87%). Additional data collected in 2023 showed that sonographers reported that additional image acquisition was requested by the interpreting physician “sometimes” (70% of responses) or “rarely” (30% of responses). Over one‐third of the sonographers (39%) thought that performing and reporting strain added a significant amount of time to their existing functional assessment workflow, but two‐thirds (65%) thought that reporting strain added value to the quality of the echo report with regards to functional assessment. The comprehensive education module, including the hands‐on training session, significantly improved LV strain knowledge for sonographers and physicians in the lab with an increase in the knowledge score from 8.8 ± 2.1 out of 16 (*n* = 59) to 11.4 ± 3.4 out of16 (*n* = 16) (*p* < 0.05).

### 3.2 Phase 3: Comprehensive Evaluation of GLS Performance, Interpretation and Reporting

From 2019 to 2023, all echocardiograms performed for CTRCD in the 4^th^ quarter of each year were included. A total of 685 consecutive CTRCD echocardiograms were reviewed for GLS performance, reporting, and accuracy (Table 2).

**TABLE 2:** GLS reporting and accuracy from 2019 to 2023.

Year	2019	2020	2021	2022	2023
**Total studies**	72	175	140	163	135
**Strain reported**	28	99	111	126	103
**Strain reported %**	39%	57%	79%	77%	76%
**Strain reported accurately**	16	60	57	100	74
**Strain reported accurately %**	57%	61%	51%	79%	72%

**FIGURE 4:**
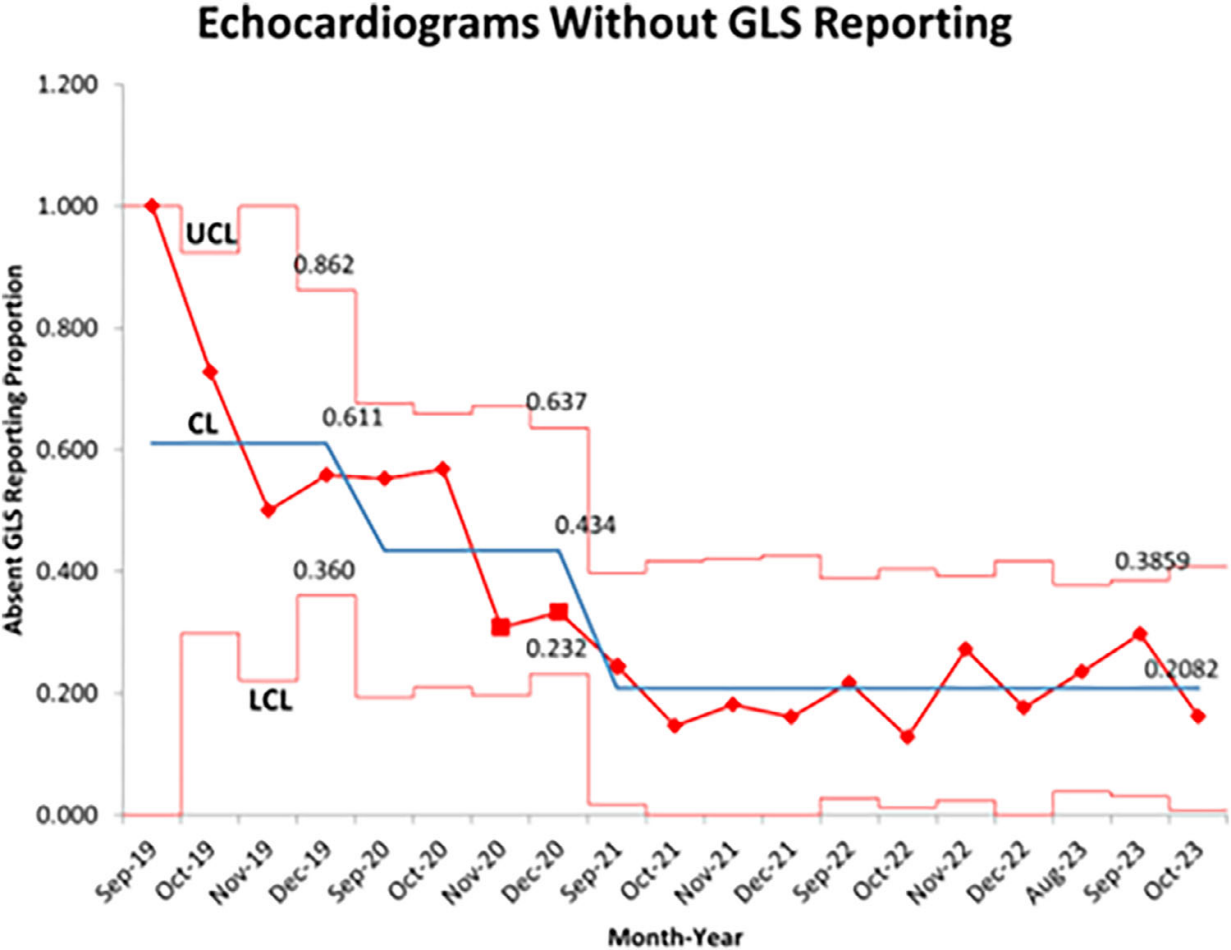
Statistical process control chart of GLS reporting throughout project displayed in p chart as proportion of non‐conforming echocardiograms without GLS (*Y*‐axis) reported by month‐year (*X*‐axis), with center line representing the average proportion of echocardiograms without GLS over time. Process shows improvement with downward shift of center line with interventions and good statistical control for final months with all data (red data points) falling between control limits. CL, center line; LCL, lower control limit; UCL, upper control limit.

**FIGURE 5:**
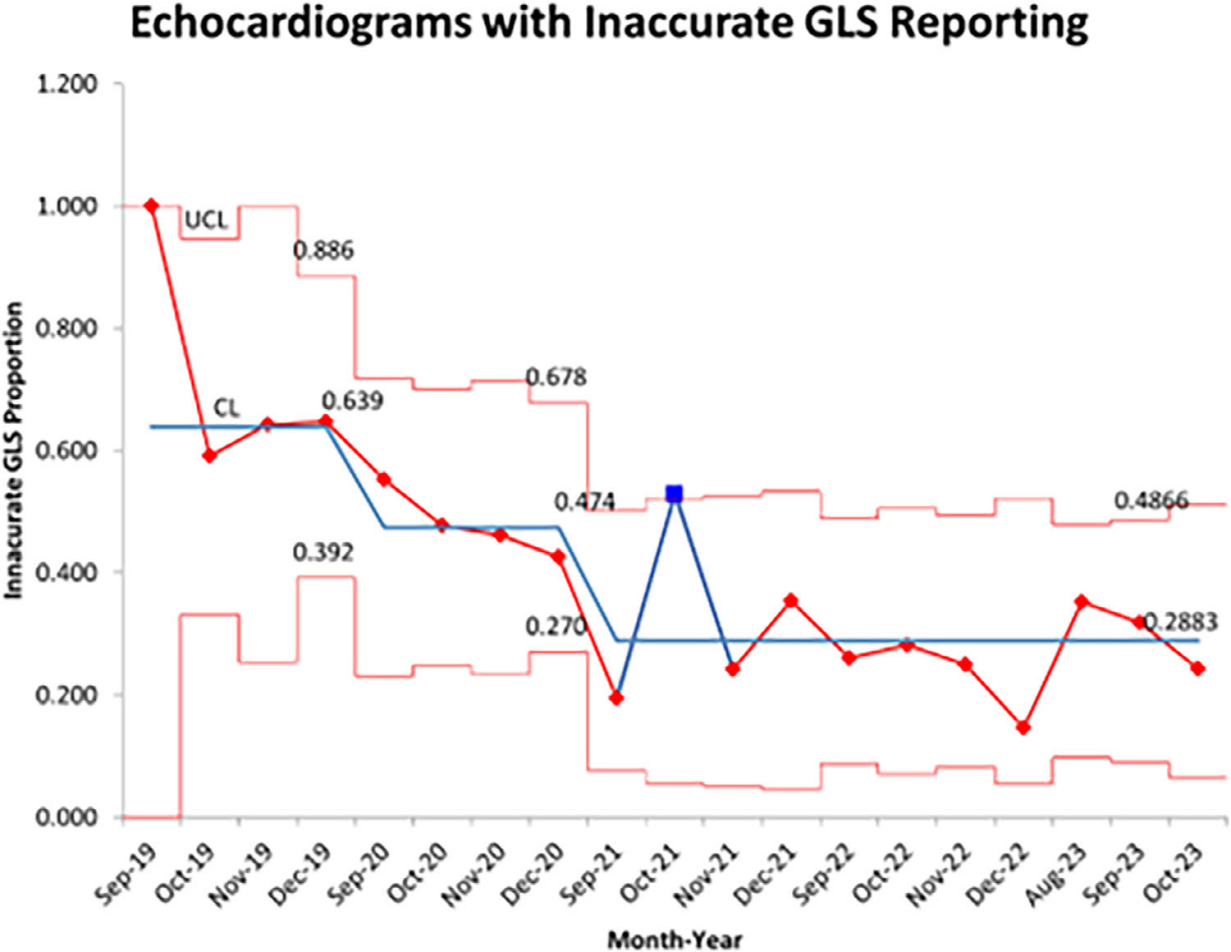
Statistical process control chart of GLS reporting accuracy throughout project displayed in p chart as proportion of non‐conforming echocardiograms without accurate GLS analysis by month‐year. Process shows overall improvement with downward shift of center line with interventions. Special cause variation noted with single data point outside control limits in October 2021. The remainder of the project demonstrated good statistical control of the process with all data falling between control limits. CL, center line; LCL, lower control limit; UCL, upper control limit.

There was an improvement in GLS accuracy from 2019 to 2023 that did not reach statistical significance (*p* = 0.09), with GLS reporting being maintained at over 75% for the last three years reviewed. Statistical process control charts (Figures 4, 5, Supplement 4) were utilized to demonstrate that GLS reporting and accuracy improved throughout the study period, overall being a stable process, with minimal special cause variation.

**FIGURE 6:**
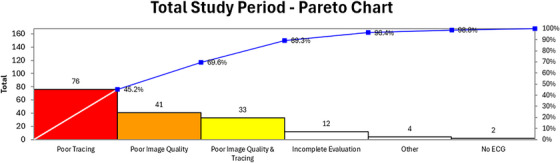
Pareto chart demonstrating the causes of inaccurate strain reporting and their relative proportion over the 5‐year study period.

A Pareto chart (Figure 6, Supplement 4) was used to evaluate the causes of inaccurate strain reporting and their relative frequency. Poor image quality and poor tracing quality independently accounted for 69.6% of the inaccurate strain reporting, with additional contribution from poor image quality and poor tracing quality together. As a result, poor image quality and poor tracing quality, either in isolation or together, accounted for almost 90% of the inaccurate strain reporting. Other causes of inaccurate reporting include no ECG tracing available, incomplete evaluation of strain images (single plane strain reporting or missing one of the required images for triplane evaluation), or other issues.

Most studies in which GLS was not reported but was measurable (99/214) did not have strain performed on the cart at time of the scan. Reporting of GLS improved from 27% in 2019 to 66% in 2023 with on‐cart automation and easier post‐processing. In addition, iterative educational endeavors led to sequential improvement in reporting and accuracy of GLS.

Overall, the number of echoes billed annually with strain increased from approximately 350 in 2018 with a denial rate of ∼96% from insurance, to about 8000 in 2023 with a denial rate of 38%.

## 4 Discussion

Implementation of strain imaging in a pediatric echo lab can be a formidable task, from initiation to ongoing maintenance of high‐quality performance and interpretation of strain imaging. Several administrative, technical and educational factors must be considered when establishing a pathway of consistent and accurate reporting of GLS in a pediatric echocardiography lab.

Our initial implementation of strain was limited due to a number of barriers including lack of strain ordering by pertinent clinicians, staff turnover, vendor differences for acquisition and analysis, time‐intensive post processing, and post‐processing software being physically remote from the primary echo reading platform. Addressing these barriers early on may streamline the implementation of strain imaging. Specifically, we found that collaboration with heart failure, nephrology and hematology‐oncology colleagues and simplifying the ordering process by incorporating strain into the electronic medical record echo order increased clinical interest and use of echocardiograms with strain imaging at our institution. The rapidly evolving technology and staff turnover called for ongoing assessment of staff comfort and skill in performing and reporting strain imaging. Investment in education for the staff on an enduring basis was necessary. To eliminate vendor differences in strain imaging, we transitioned to TOMTEC, a vendor neutral post‐processing software to allow for seamless trending of strain in our multi‐vendor lab regardless of echo machine used.

Given the initial effort we invested into implementing strain imaging in our pediatric echo lab, we anticipated higher baseline reporting and accuracy of GLS reporting when evaluating lab quality. However, when studied for quality assurance, our observed reporting and accuracy of strain reporting fell short of our expectations. This prompted us to establish a SMART aim with deliberate PDSA cycles to improve the quality of our strain imaging.

Overall, strain automation on the cart, setting optimal standards for image acquisition, integration of a vendor neutral post processing platform into the reporting software and iterative educational endeavors resulted in significant improvement in performance and reporting of accurate GLS. By incorporating these important interventions, we were able to increase strain reporting in our lab from 39% to 76% of all studies performed over a period of seven years, with sustained reporting of greater than 75% of CTRCD echocardiograms for the last 3 years.

During the initial phase of implementing GLS in our lab, 2016–2017, patients were not billed for strain performance. In 2018, once a strain order was built, echocardiograms ordered with strain imaging had an extremely high rate of denial of reimbursement from insurance at approximately 96%. This made the labor‐intensive process of performing and reporting strain an unproductive endeavor. Incorporating strain into the standard complete echo order, performing a pre‐authorization, and standardized billing with an in‐built strain CPT code led to a decrease in the denial rate to 38% allowing for commensurate reimbursement for the time and effort of the sonographers and physicians.

Similar to our findings, Ziebell et al. reported several technical and clinical factors that affect the incorporation of accurate strain into a pediatric (or any) echocardiography lab, including personnel buy‐in of newer technologies, impediments to reporting, and variability in the acquisition, performance and interpretation of GLS [15]. A practical approach to implementing strain in the adult echo laboratory, with guidance on training and quality assurance has been published [9]. A similar paper, detailing a practical approach to strain implementation, has not been reported in the pediatric literature.

There have been important efforts to prioritize and standardize image acquisition, post‐processing and interpretation to achieve inter‐vendor, inter‐software and inter‐organizational compliance [17, 19]. The importance of optimized on‐axis imaging, appropriate frame rates, clearly demarcated endocardial borders and attention to settings and algorithms in the strain software have been described [20, 21, 22, 23, 24, 25, 26]. Our results also confirmed these findings as we found that poor imaging quality and poor tracing quality, either in isolation or together accounted for almost 90% of the inaccurate strain reporting.

Despite significant improvements in inter‐vendor variability, strain algorithms and techniques vary [19, 27, 28, 29]. It is important to remember that vendor‐neutral processing software, which are separate from the echo equipment vendor still have some variance related to the underlying strain computation formula [30]. Newer studies have shown that contemporary strain software including EchoPAC and TOMTEC are able to analyze LV GLS acquired on different echo equipment vendors with adequate reproducibility [31].

To overcome these vendor differences, robust normative data has been collected in thousands of adults with slightly different lower limit of normal cutoffs depending on the platform [32, 33]. Gender based adult cutoffs using TOMTEC are similar to our institutionally agreed upon value. Pediatric normative strain studies have utilized a variety of echo machines and strain platforms which limits the compilation of robust normative data to incorporate into clinical assessment. We elected to use a strain cutoff more negative than –18% as abnormal for children > 2 years old based on institutional experience as there was limited data for use of TOMTEC endocardial strain when this cutoff was set. While a more recent study has reported strain using TOMTEC, GLS was performed in fewer than 170 children aged 1–18 and *z*‐score equations are only available for the apical 4C longitudinal strain [34]. This study and others do demonstrate age differences in strain with slightly higher absolute strain values seen in younger children before leveling off through adolescence and the importance of more robust data for *z*‐score development is clear [16, 33, 34]. Pediatric strain imaging guidelines will better guide appropriate cut‐offs for clinical practice that can be widely incorporated into daily practice. Many echo labs use echo machines from multiple vendors. We found that in such a setting, moving to a vendor neutral post processing software that is embedded into the reading platform is the most efficient way to perform and trend strain, eliminating echo machine vendor variability and improving efficiency in the workflow of the echo lab.

We believe that this internal review of our own data helped us significantly improve the quality of strain imaging and maintain strain imaging knowledge and technical skills for all members of our pediatric echocardiography lab. Our trainees have published several research papers on strain imaging, with a strong emphasis on quality strain imaging. The implementation of strain imaging requires a time commitment, both from the experts in the lab providing strain training as well as the trainees—sonographer and physicians. This time is taken away from clinical time, which can be hard for a high‐volume echo lab, especially in the setting of ongoing sonographer shortage and high turnover. Recognizing the need for an initial investment of time and effort to implement strain imaging is important, to ensure that from an institutional or organizational level, those efforts are deemed useful, with a delayed return on investment.

Our experience with the step‐by‐step approach to strain implementation as shown in the central illustration can be useful to other labs who may be in the process of implementing or optimizing GLS quality in their labs.

### 4.1 Limitations

Since there were multiple iterations of educational sessions and ongoing technical advances, our PDSA cycles did not have data timed for each intervention in terms of outcomes. Given the nature of an echo lab with ongoing staff turnover, our survey and knowledge comparisons were not made between the same group of individuals. A major limitation to widespread adoption of 2‐D speckle tracking global longitudinal strain in most echo labs is the lack of documented well‐defined reference limits for the echo platform and strain software used. Strain experts are important during the development stage, but everyday clinical use cannot be reliant on them.

## 5 Conclusions

Several administrative, technical, and educational factors must be considered when establishing a pathway of consistent, high‐quality performance and accurate reporting of GLS in a pediatric echocardiography lab. Strain automation on the cart, integration of a vendor‐neutral post‐processing platform into the reporting software, and iterative educational endeavors are essential elements of the framework. The creation of a strain taskforce or champions in a pediatric echocardiography lab ensures that sonographers and physicians quickly advance in their skills to perform, interpret, and report accurate GLS with expert supervision. Our experiences along with ongoing quality improvement processes can be used as a framework for other pediatric echo labs around the world implementing strain imaging. A similar strategy can be adopted for other forms of advanced imaging in the future.

## Funding

The authors received no specific funding for this work.

## Supporting information




**Supplement 1**: Function Protocol.
**Supplement 2**: Survey 1 ‐ Baseline comfort survey for physicians for strain imaging.
**Supplement 3**: Survey: Baseline comfort survey for sonographers for strain imaging.
**Supplement 4**:
**Figure S4**: Statistical process control chart of GLS reporting throughout project displayed in p chart as proportion of echocardiograms by month‐year without GLS reported.
**Figure S5**: Statistical process control chart of GLS reporting accuracy throughout project displayed in p chart as proportion of echocardiograms by month‐year with inaccurate GLS analysis.
**Figure S6**: Pareto chart demonstrating the causes of inaccurate strain reporting and their relative proportion over the 5‐year study period.
