# Supplementary material for: A Pathway for Improving Performance and Interpretation of Strain in a Pediatric Echocardiography Laboratory
Source: Echocardiography. 2025 Dec 11;42(12):e70369. doi: 10.1111/echo.70369 (PMC12699170; doi:10.1111/echo.70369)

Supplements:

Supplement 1: Function Protocol

**Patient selection:** To be performed on patients with known or potential ventricular dysfunction, i.e., patients with confirmed or suspected cardiomyopathy, family history of cardiomyopathy, previously documented LV dysfunction, systemic HTN or renal disease, patients with Kawasaki disease with history of significant coronary dilation or dysfunction, muscular dystrophy, metabolic disorder, previous chemotherapy, or heart transplant.

**Baseline requirements for images:**

1. Clean, large ECG tracing with visible P and QRS waves
2. Frame rates of 60-90 fps (2/3 of patient’s heart rate)
3. Acquisition of three beat loops for parasternal short axis, apical 2D and apical TDI, using a sweep speed that would incorporate at least 3 tracings.
4. Ensure apical imaging without foreshortening and on-axis planes to calculate biplane EF and for performing strain.
5. Images optimized to show the endocardium, myocardium, and epicardium with sector width wide enough to view entire myocardium throughout the cycle such that it would be traceable for strain.
6. To be able to calculate torsion the PSAX images were obtained with constant image settings from base to apex (i.e., no changes in sector width, frequency, or depth).

**Protocol:**

1. Subcostal Coronal (inverted image):
   1. Sweep from IVC/RA to RVOT 2D (post to ant – index ~ 3:00).
2. Subcostal sagittal (inverted image):
   1. Sweep from bicaval view to apex (R to L) 2D (index ~5-6:00)
   2. 3 beat clip Bicaval (SVC/IAS/IVC) 2d/color assess IVC size and collapse, PW hepatics veins.
   3. SVC 2D/Color/PW
   4. 2D/color Abdominal aorta, PW abdominal aorta
3. PLAX (index ~ 11):
   1. 3 beat clips of PLAX in 2D
   2. Zoomed AoV 2D, color - measure Ao Ann, Ao Rt, Ao STJ, Asc Ao
   3. Zoomed MV 2D, color.
   4. RVIF (TV) 2D/color, CW for TR jet- measure TR jet of able
   5. RVOT (PV) 2D/color, CW PI – PI jet if able
4. PSAX (index ~ 2):
   1. Sweep from Base (AoV) to Apex: 2D.
   2. 3 beat clips of PSAX 2D at base level
   3. RVOT: 2D/color. PW RVOT, CW PV for PI
   4. TV: 2D/color. CW TR jet
   5. 3 beat clips at MV level in LV: 2D/color
   6. 3 beat clips at LV pap level & Apex: 2D – Measure LV
5. Apical:
   1. 3 beat 2D clip of **A4C**
      1. Focused LV/MV/LA
         1. LA– 2D, LA volume/strain, Pulmonary vein 2D/color/PW (at least 1 vein)
         2. MV – 2D/color, MV leaflet tips – PW/CW, Measure MV annulus, MV E/A
         3. Focused LV – 2D (measure Biplane + LV strain)
      2. Focused RV/TV/RA
         1. RA – RA area
         2. TV - 2D/color, TV leaflet tips – PW/CW, TR, Measure TV annulus, TV E/A
         3. RV – FAC, RV strain)
      3. TDI – MV lat, MV med, TV free wall, TAPSE
   2. 3 beat 2D clip of **A3C** (rotate clockwise till AoV opens)
      1. Focused LV 2D (for strain)
      2. 2D/color LVOT/AoV
      3. PW LVOT & AOV. CW AoV
   3. 3 beat 2D clip of **A2C** (rotate clockwise till AoV is no longer visual)
      1. LV focused view (measure Biplane + strain)
      2. LA focused view (LA volume)

**Measurements:**

| Window | 2D | Doppler Measurement |
| --- | --- | --- |
| PLAX | Ao Ann, Ao Rt, Ao STJ, Asc Ao | TR jet  PI jet |
| PSAX | LV 2D measurements | CW PI jet  TR Jet |
| Apicals | LA Volume, Strain  LV Volume, Simpsons, Strain  RV Volume, RV strain  RA area  MV annulus  TV annulus | MV E/A  TV E/A  TR jet  PW LVOT  CW AoV  TDI LV lat, med & RV TV  TAPSE |

Abbreviations: 2D – 2 dimensional, A2C – apical two chamber, A4C apical four chamber, Ann annulus, AoV aortic valve, Asc Ao ascending aorta, CW continuous wave, ECG electrocardiogram, EF ejection fraction, FAC – fractional area change, Fps frames per second, HTN hypertension, IVC inferior venacava, LA left atrium, LV - Left ventricle LVOT left ventricular outflow tract, MV mitral valve, PLAX parasternal long axis, PI pulmonary insufficiency, PSAX – parasternal short axis PW pulse wave, RA right atrium, RV right ventricle, RVIF right ventricle inflow, RVOT right ventricular outflow tract, STj ST junction, SVC superior vena cava, TAPSE tricuspid annular plane systolic excursion, TDI – tissue Doppler imaging, TR tricuspid regurgitation

Supplement 2. Survey 1 – Baseline comfort survey for physicians for strain imaging

Survey

1. How often do you review to make sure that a complete function study includes all the following: systolic & diastolic function assessment, tissue Doppler, strain, and 3D EF?

Never/ rarely/ sometimes /most of the time/ Every time

1. On a scale of 1-5, how comfortable are you in identifying whether strain imaging was performed and post-processed?

Not comfortable (1) /Rarely comfortable/Somewhat comfortable/Mostly comfortable/Very comfortable (5)

1. On a scale of 1-5, how comfortable do you feel in evaluating whether adequate strain contouring was performed by the sonographer?

Not comfortable (1) /Rarely comfortable/Somewhat comfortable/Mostly comfortable/Very comfortable (5)

1. Can you identify a normal strain curve?

No /I think so/ Yes.

1. Do you know what are considered normal global longitudinal strain values in adults?

No /I think so/ Yes.

1. Do you know how to post-process strain images if you are not satisfied with the post-processing done by the sonographer?

No /I think so/ Yes.

1. Do you know where to report strain values in the echo report?

No/I think so/ Yes.

1. How often do you send the sonographer back to the patient room to acquire more images for strain assessment? Never/ rarely/ sometimes /most of the time/ Every time
2. Do you think that reporting strain adds a significant amount of time to your existing reporting time for echoes done to assess function? No /I think so/ Yes.
3. Do you think that reporting strain adds value/improves the quality of your echo report on function assessment?

Never/ rarely/ sometimes /most of the time/ Every time

Supplement 3. Survey: Baseline comfort survey for sonographers for strain imaging

1. Based on the echo indication, do you think you recognize that a patient needs a complete function study including strain and 3D EF?

Never/ rarely/ sometimes /most of the time/ Every time

1. Overall, how comfortable are you in acquiring images for strain on the GE machine?

Not comfortable /Rarely comfortable/Somewhat comfortable/Mostly comfortable/Very comfortable

1. Overall, how comfortable are you in acquiring images for 3D EF on the GE machine?

Not comfortable /Rarely comfortable/Somewhat comfortable/Mostly comfortable/Very comfortable

1. How often do you check to see if the strain images track appropriately?

Never/ rarely/ sometimes /most of the time/ Every time

1. How often do you check to see if the 3D EF images track appropriately?

Never/ rarely/ sometimes /most of the time/ Every time

1. Can you identify a normal strain curve?

No /I think so/ Yes.

1. Do you know what are considered normal global longitudinal strain values?

No /I think so/ Yes.

1. Do you know how to post-process strain images on the Echo PAC workstation?

No /I think so/ Yes.

1. Do you understand the concept of strain imaging?

No /I think so/ Yes.

1. How often do you think the physician send the sonographers back to the patient room to acquire more images?
2. Do you think that performing and reporting strain adds significant time to your required time for function assessment?
3. Do you think that reporting strain adds value/improves the quality of your echo report on function assessment?

Never/ rarely/ sometimes /most of the time/ Every time

Supplement 4

Statistical process control charts were utilized to demonstrate GLS reporting and GLS accuracy throughout the study period. Due to the nature of the data, p charts were chosen to display the proportion of studies not reporting GLS or with inaccurate GLS analysis. GLS reporting increased over the study period, as demonstrated by the p chart, with an equivalent decrease in the proportion of studies without GLS reported (Figure 4). There was an appropriate downward shift of the center line with all data points between the upper and lower limits suggesting a stable process with no special cause variation.

Figure 4: Statistical process control chart of GLS reporting throughout project displayed in p chart as proportion of echocardiograms by month-year without GLS reported.


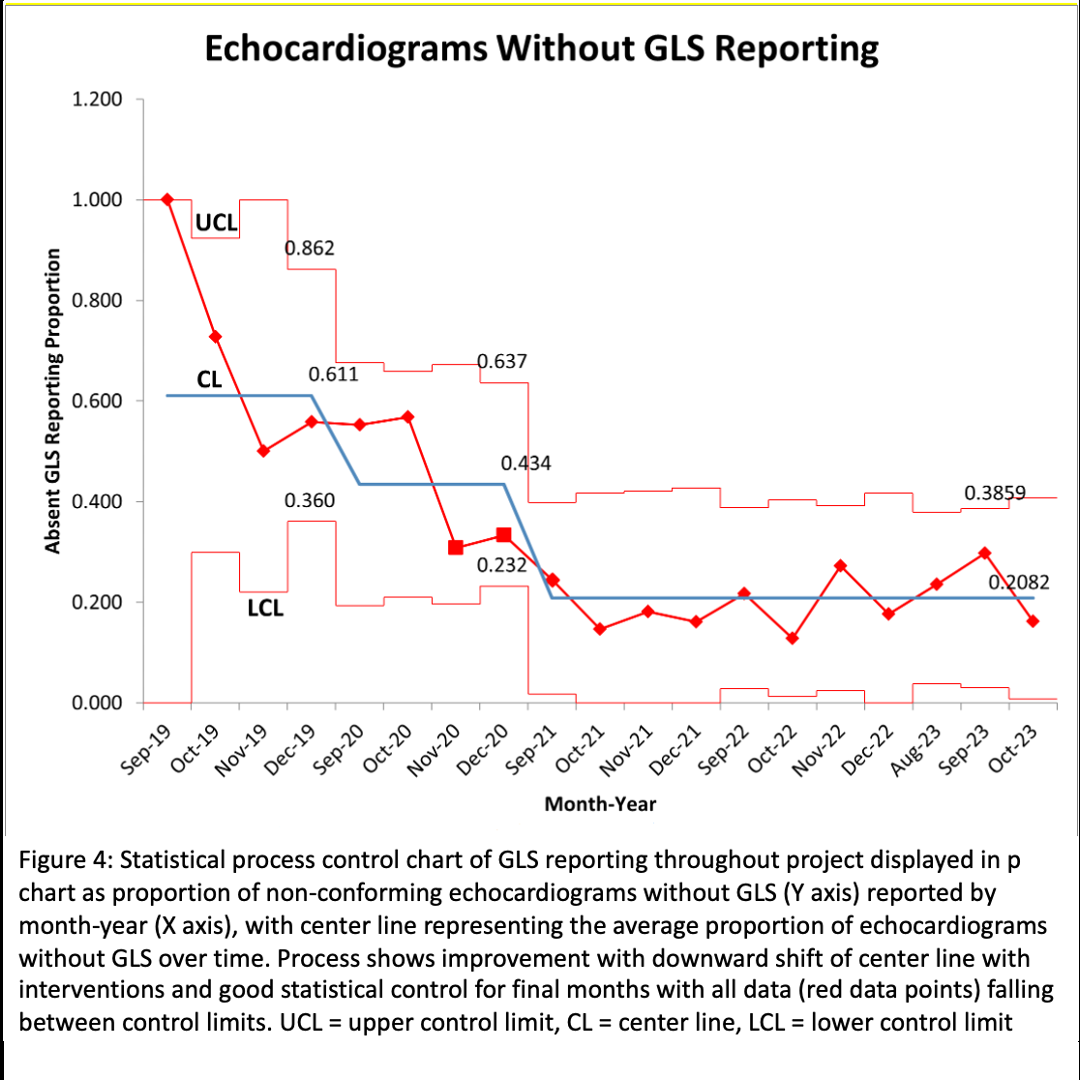


Accuracy of GLS also increased, as demonstrated by the p chart, with an equivalent decrease in proportion of studies with inaccurate GLS (Figure 5). There was an appropriate downward shift of the center line with most data points between the upper and lower limits with only one data point in October 2021 with a special cause variation.

Figure 5: Statistical process control chart of GLS reporting accuracy throughout project displayed in p chart as proportion of echocardiograms by month-year with inaccurate GLS analysis.


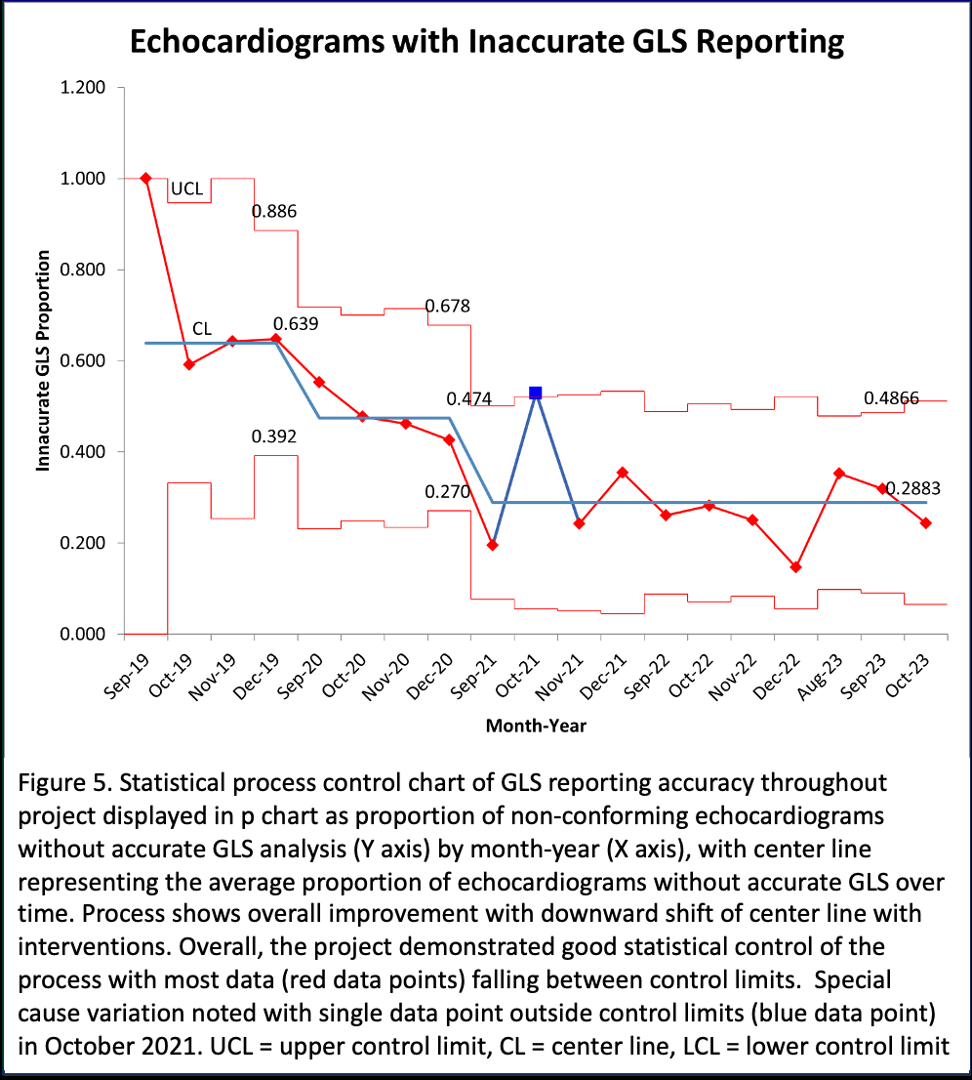


A pareto chart was used to evaluate the causes of inaccurate strain reporting and their relative frequency. Poor image quality and poor tracing quality independently accounted for 69.6% of the inaccurate strain reporting, with additional contribution from poor image quality and poor tracing quality together. As a result, poor image quality and poor tracing quality, either in isolation or together, accounted for almost 90% of the inaccurate strain reporting (Figure 6). Other causes of inaccurate reporting include no ECG tracing available, incomplete evaluation of strain images (single plane strain reporting or missing one of the required images for triplane evaluation), or other issues.

Figure 6: Pareto chart demonstrating the causes of inaccurate strain reporting and their relative proportion over the 5-year study period.


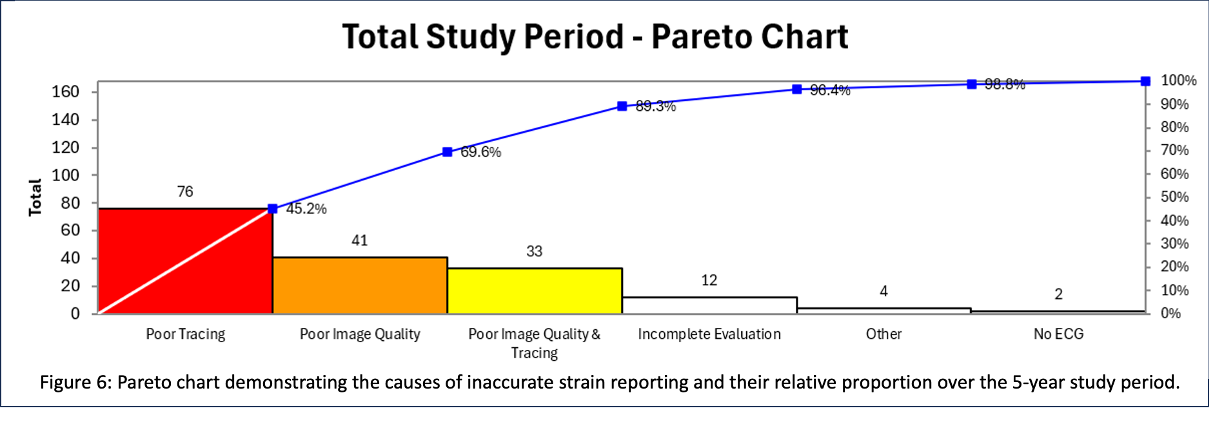

Supplement: Supplementary file 1 — Supplement 1: Function Protocol. Supplement 2: Survey 1 ‐ Baseline comfort survey for physicians for strain imaging. Supplement 3: Survey: Baseline comfort survey for sonographers for strain imaging. Supplement 4: Figure S4: Statistical process control chart of GLS reporting throughout project displayed in p chart as proportion of echocardiograms by month‐year without GLS reported. Figure S5: Statistical process control chart of GLS reporting accuracy throughout project displayed in p chart as proportion of echocardiograms by month‐year with inaccurate GLS analysis. Figure S6: Pareto chart demonstrating the causes of inaccurate strain reporting and their relative proportion over the 5‐year study period. [file ECHO-42-e70369-s001.docx]
